# Comparison Between Surgical and Percutaneous Paddles in Spinal Cord Stimulation for Chronic Neuropathic Pain

**DOI:** 10.3390/jcm14197013

**Published:** 2025-10-03

**Authors:** Marta Antonia Gómez-González, Nicolás Cordero-Tous, Carlos Sánchez-Corral, Beatriz Lechuga-Carrasco, Manuel Alejandro Sánchez-García, Rafael Gálvez-Mateos, Gonzalo Olivares-Granados

**Affiliations:** 1 Functional Neurosurgery Unit, Department of Neurosurgery, Hospital Universitario Virgen de las Nieves, Juan Pablo II Avenue, S/N, 4th Floor, 18013 Granada, Spain; martaa.gomez.sspa@juntadeandalucia.es (M.A.G.-G.); carlos.sanchez.corral.sspa@juntadeandalucia.es (C.S.-C.); beatriz.lechuga.sspa@juntadeandalucia.es (B.L.-C.); gonzalo.olivares.sspa@juntadeandalucia.es (G.O.-G.); 2 Pain Unit, Department of Anesthesiology, Reanimation and Pain Management, Hospital Universitario Virgen de las Nieves, Juan Pablo II Avenue, S/N, 2nd Floor, 18013 Granada, Spain; manuel.sanchez@ugr.es (M.A.S.-G.); rmergalz@ugr.es (R.G.-M.); 3 Department of Anatomy, University of Granada, Doctor Jesus Candel Fabregas Avenue 11, 18016 Granada, Spain

**Keywords:** pain surgery, neuropathic pain, chronic pain, failed back syndrome, complex regional pain syndrome

## Abstract

**Background**: Spinal cord stimulation (SCS) is a well-established treatment for chronic neuropathic pain, offering a safe procedure with low complication rates. Both surgical and percutaneous leads can be effective, with similar complication rates. **Methods**: We analyzed all patients implanted at a reference center since 1996 to compare pain control and complications and determine whether one system was more effective than the other in patients who had experienced both systems. A retrospective observational study was designed. **Results**: A total of 188 SCS systems were implanted, with a follow-up period of 79.71 ± 60.39 months (mean ± SD). We analyzed data from 106 males (56.38%) and 82 females (43.62%), ranging from 15 to 76 years old. A total of 68 (36.17%) surgical leads and 120 (63.83%) percutaneous leads were implanted for failed back syndrome (120, 63.83%), complex regional pain syndrome (56, 29.79%), and other conditions (12, 6.38%). No statistically significant differences were found in any variables except for lead migration (*p* = 0.05). In patients who initially had a percutaneous system and later received a surgical system, we found a statistically significant difference in pain relief percentage (*p* = 0.03) and a trend toward statistical significance in the PGI-C score (*p* = 0.08). **Conclusions**: Both surgical and percutaneous leads have demonstrated similar pain control rates, but percutaneous leads have a higher migration rate. Complications can be minimized by performing the procedure in specialized centers with extensive experience. Further studies comparing both systems should be conducted to determine if one type of lead is superior.

## 1. Introduction

The International Association for the Study of Pain (IASP) defines pain as an unpleasant sensory and emotional experience associated with, or resembling that associated with, actual or potential tissue damage [1]. Pain represents a warning about tissue damage signaled by specific receptors and fiber systems [2]. However, some patients develop neuropathic pain, which is defined by the IASP as being caused by a lesion or disease of the somatosensory nervous system [3]. Prevalence of neuropathic pain can be as high as 7–10% [4,5], accounting for 20 to 25% of individuals with chronic pain [6]. There is no gold standard to diagnose neuropathic pain [2], so the diagnosis is based on clinical criteria [6]. Current treatment guidelines include pharmacological treatment, neurostimulation, multimodal approaches and psychotherapy [5].

Spinal cord stimulation (SCS) is a well-established treatment for chronic neuropathic pain, including failed back syndrome (FBS) and complex regional pain syndrome (CRPS) [7,8]. It is considered a safe procedure, with low complication rates reported in the literature [7,9,10,11], such as lead migration (13.2%), infections (3.4%), epidural hemorrhage (0%), pain over the implant site (0.9%), pocket problems (9.1%) and hardware malfunction (2.9%) [11]. These complications can occur at implantation, such as epidural hemorrhage, or over time, such as lead migration, infections, pain over the implant site, pocket problems and hardware malfunction. In addition, SCS has been shown to be more effective than conservative management alone in multiple studies [11,12], so it is widely used and recommended as a second-line therapy in patients with chronic, neuropathic pain that does not respond to pharmacological treatment [5]. Based on neuromodulation of the dorsal column, SCS allows for the adjustment of stimulation intensities over the course of the underlying disease [13].

When first introduced in 1967 [14], only surgical leads were available, requiring general anesthesia and a laminectomy for implantation. Later, in 1975, percutaneous leads were developed to avoid general anesthesia, allowing implantation under local anesthesia in an outpatient setting [14]. This enables patients to remain awake during the procedure, providing real-time feedback to the surgeon to ensure stimulation coverage of the painful area.

Both systems are widely used, with a growing preference for percutaneous implantation [15]. Some studies have compared the two approaches, showing similar pain relief outcomes, although surgical leads are associated with a higher rate of implant-related complications [7,10,13,16,17,18,19], like spinal cord injury, with an incidence reported of 2.13% [10], and implant-related complications, with an incidence of up to 9.4% [7]. However, the literature on the implantation of one system following the failure of the other is scarce, mostly limited to case reports [20,21,22].

With this in mind, we present a retrospective study analyzing all patients implanted with an SCS system at Hospital Virgen de las Nieves (Granada, Spain) since 1996, as a reference center. The primary objective is to compare pain relief and complications between surgical and percutaneous leads. The secondary objective is to evaluate whether patients who received both systems achieved better pain control with one system over the other.

## 2. Materials and Methods

After obtaining regional approval from the Ethics Committee (study NC-D-01, Ethics Committee reference SICEIA-2020-000438), we conducted a retrospective review of all patients who received an SCS implant at Hospital Virgen de las Nieves (Granada, Spain) from November 1996 to December 2023.

**Figure 1:**
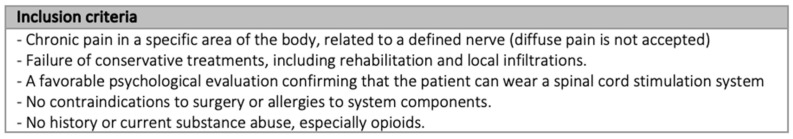
Inclusion criteria for spinal cord stimulation. Adapted from Gómez-González et al., 2025 [24].

In our center, a multidisciplinary committee composed of neurosurgeons, neurologists, neurophysiologists, anesthesiologists and rehabilitation doctors evaluates each case proposed for SCS. To be a candidate, the patient needs to have chronic pain in a specific area of the body, with failure of other treatments, and no contraindications to surgery. The inclusion criteria for eligibility to receive a spinal cord stimulation system are further explained in Figure 1. To assess psychological status, every patient is evaluated by a neuropsychologist, who evaluates if the patient has any psychological condition that could contraindicate implantation, such as personality disorders or substance dependence.

After approval for SCS system implantation, the procedure is performed in two phases for both surgical and percutaneous leads.

First, a trial phase is conducted, in which one or more leads are implanted in the epidural space and connected to an external stimulator. Effectiveness is assessed over a four-week period using the verbal numerical rating score (VNRS). The trial is considered successful if the patient reports at least 50.00% pain relief. If this threshold is met, the implantation phase follows, during which a permanent stimulator is placed in the adipose tissue of the abdomen or lower back. If the trial is unsuccessful, the system is completely removed.

The primary difference between surgical and percutaneous lead lies in the implantation process. Surgical leads require general anesthesia, with the lead placed at the upper side of D8 for FBS and at C2 for CRPS. However, there is no intraoperative monitoring to confirm that the lead covers the pain area. Percutaneous leads, on the other hand, are implanted under local anesthesia, following the same anatomical landmarks. A paresthesia-based stimulation technique is used to confirm adequate pain coverage, allowing for real-time lead adjustments to optimize pain relief.

In every intervention, the same antibiotic prophylaxis is administered 30 min before the skin incision, using a single dose of 2 g of cefazoline or 800 mg of teicoplanin according to our hospital guidelines. If there is a high risk of infection, we continue this prophylaxis for 24 h. Patients have a consultation with a specialized nurse a week after implantation, to detect early infection; and patients have a mobile phone app with a support center that they can use if they have any problems.

When a superficial infection occurs, and the system is effective, treatment consists of empirical oral antibiotics (as this is a post-operative infection, those that cover Gram-positive cocci are used following our hospital’s preventive medicine protocol), taking a sample of the infection if possible, and with antibiotics adjusted to the antibiogram until the infection is fully resolved. We do not consider removing the system the first time an infection occurs, as we consider that prolonged antibiotics and are enough, and patients are dependent on the SCS system to control pain. We only remove the system when it does not provide pain relief, because the system is not needed, or when it is the second infection in the same patient, as it has not been cured with the best antibiotic treatment.

When a percutaneous system that successfully relieves pain needs to be removed due to system failure and/or infection, approximately two months after the infection has been cleared, the case is reviewed by a multidisciplinary committee. Upon approval for reimplantation, a surgical system is selected. This approach is preferred due to the potential technical challenges associated with reimplantation, which may arise from inflammatory changes induced by the prior infection, as previously reported in the literature [20,22].

For each implanted lead, we collected demographic data at the time of implantation, as well as details on electrode type, implantation site, type of stimulation, number of generators, and complications. We also recorded the number of subsequent surgeries required due to complications. Age was stratified into two groups, using 40 years as the cutoff, based on previous studies that have identified significant differences in chronic pain prevalence and quality of life between age groups [23]. Complications were categorized as minor or major, based on severity, as detailed in Table 1.

**Table 1:** Classification of complications.

Major Complications	Minor Complications
Infection	Unwanted electric pulse
Lead migration	Pocket problems
Epidural hematoma	Impedance increase

**Figure 2:**
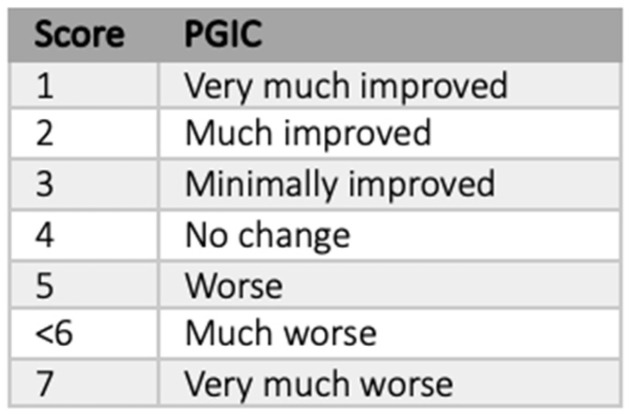
Patient Global Impression of Change. Adapted from Dworkin et al., 2008 [25].

To assess clinical status, we used the Patient Global Impression of Change (PGI-C), shown below in Figure 2, the percentage of pain relief achieved, and whether they would get implanted again. The PGI-C evaluates subjectively the changes perceived by the patient, ranging from very much improved to very much worse. We considered that the SCS system was effective if there was a minimal improvement or if a pain relief of 50% was achieved. To obtain this data, since 2020, we have developed a remote follow-up system, so patients implanted from that date answer these data with a mobile phone app [24]. For all patients implanted before 2020, these scales were answered in their last consultation. We only analyzed clinical data obtained from these scales, assuming those who did not answer as a loss.

In patients who had percutaneous lead implanted, it was removed, and then surgical lead was implanted. We registered the reason for the explanation, and we asked patients to provide answers for the clinical scales regarding both systems.

For data analysis, we used SPSS^®^ 20.0 (IBM; Armonk, NY, USA) to compare the data collected between percutaneous and surgical leads. The *t*-Student test for independent samples was used to compare age, while the Mann–Whitney U test and Fisher’s exact test were applied to analyze the other variables. The correlation between PGI-C score and percentage of pain relief achieved was assessed using Spearman’s correlation coefficient.

Some patients initially received a percutaneous lead, followed by the implantation of surgical lead. In these cases, we performed a subgroup analysis using the Wilcoxon test to compare PGI-C scores and the percentage of pain relief achieved.

Similarly, some patients were initially implanted with tonic stimulation but later switched to high-frequency stimulation, experiencing better outcomes. Additionally, some patients did not achieve satisfactory pain relief with tonic stimulation but did with high-frequency stimulation. This aspect was analyzed in a previous study [26]; therefore, it was not further examined in the present study.

## 3. Results

### 3.1. Epidemiological Data

From November 1996 to December 2023, a total of 188 SCS systems were implanted, with 106 male patients (56.38%). The majority of patients (153, 81.38%) were 40 years old or older. The mean follow-up period was 79.71 ± 60.39 months, with a maximum follow-up of 321 months.

Regarding the cause of pain, we identified 120 patients (63.83%) with failed back syndrome (FBS) and radiating pain, 56 (29.79%) with complex regional pain syndrome (CRPS), and 12 (6.38%) with other pain etiologies.

A total of 120 patients (63.83%) received a percutaneous lead, while 68 (36.17%) were implanted with surgical lead. In terms of spinal placement, 36 leads (19.15%) were cervical, whereas 152 (80.85%) were thoracic.

The most common stimulation type was tonic stimulation, used in 167 patients (88.83%), with the remainder receiving high-frequency stimulation. However, among the 167 patients with tonic stimulation, 14 (7.45%) later transitioned to high-frequency stimulation. All patients who undergo high-frequency stimulation have percutaneous leads. This information is detailed in Table 2.

Regarding complications, 28 patients (14.99%) experienced at least one major complication, with a total of 36 major complications recorded. Among them, 22 patients had a single major complication, while six patients had two major complications. The most common major complications were infection (19 cases, 52.78%), lead migration (15 cases, 41.67%), and epidural hematoma (3 cases, 5.56%). This means that 19 out of the 188 patients had an infection, representing 10.11% of the total. Among the 19 patients with infections, 10 patients (52.63%) had a single infection, 4 patients (21.05%) had two infections, 1 patient (5.26%) had three infections, and 4 patients (21.05%) had more than three infections. In patients with more than one infection, the same microorganism was isolated every time, so we consider it a chronic infection.

Regarding minor complications, a total of 68 minor complications were observed in 58 patients (30.85%). This represents 36.17% of the 188 patients included. Referring to minor complications, 38 patients had a single minor complication, while the remaining 20 patients had two minor complications. Additionally, 19 patients (10.01%) experienced both a minor and a major complication. The most common minor complications were pocket-related issues (36 cases, 52.8%), increased impedance (24 cases, 35.29%) and unwanted electrical pulses (8 cases, 11.76%). This information is summarized in Table 3.

To address both major and minor complications, 57 out of the 86 patients (66.28%) who experienced any complication required surgical intervention. Among them, 38 patients (66.67%) underwent one revision surgery, 12 patients (21.05%) required two surgeries, and 7 patients (12.28%) underwent three or more surgeries. In total, 89 surgical procedures were performed to manage these complications.

A total of 26 patients (13.82%) were lost to follow-up due to reasons unrelated to the SCS system (3 deaths, 4 cases of mental impairment, 9 patients relocating, and 10 discontinuing follow-up). This left 162 patients who completed the clinical survey, and this dataset was analyzed to assess clinical outcomes.

Regarding employment status at the time of implantation, 18 patients (11.11%) were actively working, 47 patients (29%) were on temporary leave, and 97 patients (59.88%) were either unemployed or on permanent leave due to health conditions. Among the 47 patients on temporary leave, 10 (6.17%) were able to return to work, including 8 patients (4.24%) who were over 40 years old.

Based on the PGI-C scale, the SCS system was deemed effective in 140 patients (86.42%), and 91 patients (56.17%) achieved ≥50% pain relief, meeting the threshold for clinical efficacy. Additionally, 131 patients (80.86%) reported that they would undergo the implantation again. This data is summarized in Table 4.

Using Spearman’s correlation between the PGI-C scale score and pain relief percentage, we found a correlation coefficient of 0.79 (*p* < 0.001). This result is consistent, as a lower PGI-C score indicates greater overall improvement, which aligns with higher pain relief.

**Figure 3:**
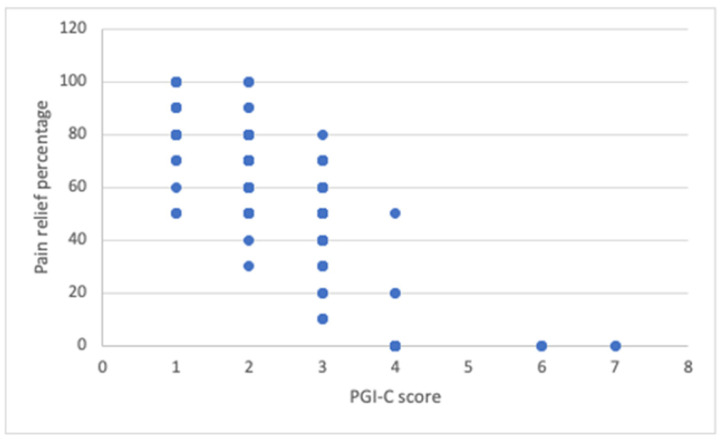
Kappa correlation coefficient analysis between the effectiveness of the SCS system in pain control and the PGI-C scale.

When comparing the effectiveness of the SCS system using both scales, we obtained a Kappa correlation coefficient of 0.34, indicating a fair agreement between the two measures. This data is illustrated in Figure 3.

### 3.2. Comparison Between Surgical and Percutaneous Leads

**Figure 4:**
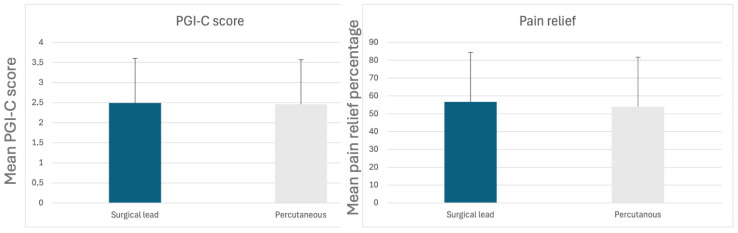
Comparison between surgical and percutaneous leads in pain relief.

Comparing surgical and percutaneous leads, we found no statistically significant differences in the number of complications (*p* = 0.324), PGI-C score (*p* = 0.887) and pain relief (*p* = 0.302). Among the 19 cases of infection, 10 occurred in surgical leads and 9 in percutaneous leads, showing no significant difference (*p* = 0.304). However, when analyzing complications, we found a statistically significant difference in lead migration (*p* = 0.056), whereas differences in other complications were not statistically significant. All compared variables are summarized in Table 5, Table 6 and Table 7 and illustrated in Figure 4.

### 3.3. Subgroup Analysis

**Figure 5:**
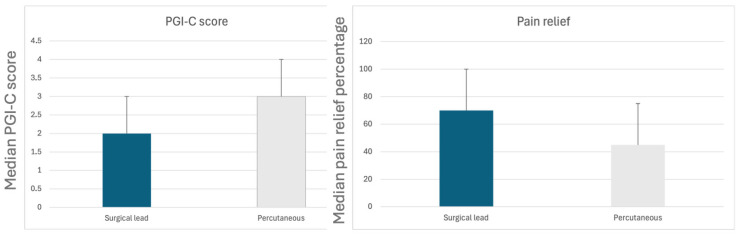
Clinical comparison between surgical and percutaneous leads in patients who have had both systems.

We analyzed ten patients who were initially implanted with percutaneous leads and later switched to surgical leads, using the Wilcoxon test. The reasons for conversion included system failure (*n* = 5), lead migration (*n* = 4) and infection (*n* = 1). Our analysis revealed a statistically significant improvement in pain relief percentage (*p* = 0.033) and a trend toward statistical significance in PGI-C score (*p* = 0.084). These findings are summarized in Table 8 and illustrated in Figure 5.

## 4. Discussion

In recent years, the number of implantations of SCS systems has increased due to their proven effectiveness. Between 2009 and 2018, percutaneous lead placement rose by 252%, while paddle lead placement increased by 142%, a trend first noted in 2000 [15]. This growth is largely attributed to cost reduction and the advantages of minimally invasive techniques. A recent study confirms this trend, showing that patients discharged the same day were mostly implanted with percutaneous leads [27]. In our center, we have used both lead types since 2000, but since 2015, we have favored percutaneous leads to enable same-day discharge, reserving surgical leads for cases where percutaneous systems fail. For this reason, all patients who use high-frequency stimulation have percutaneous leads.

Manchikanti et al. [15] highlighted that the indications for SCS have expanded due to its effectiveness. While this study did not analyze implantation site differences, we found a statistically significant distinction between surgical and percutaneous leads, with surgical leads more frequently placed at dorsal levels. This shift aligns with the increase in complex regional pain syndrome (CRPS) cases referred to for SCS after 2015.

The first comparative study of percutaneous and surgical leads was published in 2000 by Villavicencio et al. [19], analyzing 41 patients—15 (56%) with percutaneous leads and 12 (44%) with surgical leads. Both groups experienced significant initial pain reduction (VAS score), but surgical leads provided greater long-term relief (*p* = 0.02). Later, Kinfe et al. [13] assigned 100 failed back syndrome (FBS) patients to either cylindrical or paddle lead implantation in a nonrandomized manner. They found similar pain relief at a one-year follow-up, but as paddle leads were placed at T7-T8 and cylindrical leads at T10-T11, this may have introduced bias by comparing leads positioned at different spinal levels.

In 2022, Beletsky et al. [16] analyzed national data from the Healthcare Corporation of America, reviewing 9935 SCS patients implanted between 2015 and 2020. The most common indications for SCS in their study were chronic pain (*n* = 6124, 61.6%), post-laminectomy syndrome (*n* = 4411, 44.4%), and neuritis (*n* = 3747, 37.7%). Unlike our study, in which post-laminectomy syndrome was the primary indication, their population had a broader range of pain conditions.

Beletsky et al. [16] also reported that open surgical placement was associated with longer hospital stays (0.58 ± 0.76 vs. 0.36 ± 0.64 days, *p* < 0.001). We did not analyze hospital stay length, but in our center, all patients with paddle leads remain hospitalized for 1–2 days, while those with percutaneous leads are discharged the same day. Though we lack objective data, our findings align with Beletsky et al. [10] and are further supported by Spirollari et al. [7].

Regarding complications, Beletsky et al. [16] found significant differences between open and percutaneous approaches, with percutaneous implants associated with a higher overall complication rate (open: 0.15 ± 0.44, percutaneous: 0.26 ± 0.53, *p* = 0.04). Our study found no significant differences between the two groups except for lead migration (open: 2.94%, percutaneous: 10.83%, *p* = 0.051), a complication previously reported to be more common in percutaneous leads [20,21,28].

Spirollari et al. [7] analyzed the National Inpatient Sample and found that open SCS placement was significantly associated with implant-related complications (OR = 3.247, CI 1.9–5.55, *p* < 0.001) at initial implantation or full system replacement. Other than implant complications, no significant differences were found. Our findings differ in that we did not observe increased implant-related complications in open procedures, with the only statistically significant difference being lead migration, which was higher in percutaneous placements. We believe this discrepancy could be due to our experience as a reference center, performing SCS implantation for over 20 years, which may contribute to minimizing complications in both techniques.

In a recent metanalysis [29], the infection in patients with surgical leads was estimated at 0.050 (95% CI [0.037, 0.066]), with percutaneous leads being estimated at 0.033 (95% CI [0.028, 0.040]), which was statistically significant (*p* < 0.0001). In our study, we found no statistically significant difference between the two groups (surgical leads 12, 17.65%; percutaneous leads 22, 18.33%; *p* = 0.278). This same metanalysis [29] states that they found a statistically significant difference in lead migration, with percutaneous leads migrating more than paddle leads (proportion 0.072 vs. 0.043, respectively, *p* = 0.011). This is consistent with our findings.

Regarding the number of infections, we had a total of 19 patients with infections (52.78% of major complications, 10.11% of the total population), which is a bit higher than previous rates reported of 3.4% by Cameron et al. [11]. We believe this is because we included superficial cutaneous infection in the pocket, whereas, in that article, the site of infection was not clearly specified. In the same fashion, infection at the surgical site was not analyzed in a recent comparison [7]. This leads us to believe that superficial infection related to the pocket is underestimated, and the incidence is not well known. In a recent metanalysis, it is specified that minor issues occur in 30–40% of patients [29] and that they resolve with time. In our group, we found 68 minor complications, representing 36.17% of the 188 patients included, so we believe our results in that regard are similar.

In our subgroup analysis, we found no previous studies comparing pain relief in patients who received paddle leads after the failure of cylindrical leads. The first case report of paddle lead implantation after percutaneous lead migration was published in 2019, demonstrating good clinical response [14]. In 2020, another case reported successful pain control after implanting a paddle lead alongside a cylindrical lead [21]. To our knowledge, this is the first case series comparing pain relief between cylindrical and paddle leads in the same patients. In our study, pain relief improved significantly from 45 ± 30% with percutaneous leads to 70 ± 30% with surgical leads (*p* = 0.035). Although our sample size is small, these promising results suggest that this approach warrants further investigation.

Comparing our results with current literature, in general, we believe our findings are supported by previous studies. Even if current trends go in favor of percutaneous paddles [27], our study suggests that both percutaneous and surgical paddles have the same rates of complications except for paddle migration. We believe that being a reference center has helped us minimize complications, thus presenting surgical paddles as a safe alternative with a lower migration rate.

### Limitations

This study has a few limitations. First, as a retrospective analysis with patients from a single center, our sample size is relatively small, particularly in the subgroup analysis of patients who switched from percutaneous to surgical leads. However, we have not found a larger single-center case series in the literature. Second, our study spans a long period, with the first case in 1996. Since then, there have been significant advancements in hardware and stimulation techniques, making direct comparisons challenging. Earlier, patients only had surgical paddles, whereas since 2015, we have tended to implant percutaneous paddles. This can make comparisons difficult. Additionally, we acknowledge a recall bias in the subgroup analysis, as pain relief was assessed when the patient had already transitioned to the surgical lead. In addition, the subgroup is composed of only 10 patients, so no recommendations should be made due to a lack of statistical power.

Another limitation is that in our center, open surgical implantation is performed under general anesthesia, while percutaneous leads are implanted under local anesthesia. The associated costs, including hospitalization and procedural differences, were not analyzed. Moreover, percutaneous lead placement allows real-time feedback from awake patients, confirming paresthesia coverage of the pain region, whereas surgical implantation does not offer this advantage.

To better compare these two implantation techniques, prospective studies are needed, ideally with standardized pain assessment methods and cost analysis. Further research should also explore the benefits of paddle leads after percutaneous system failure in a larger patient population.

## 5. Conclusions

Surgical and percutaneous leads used in SCS provide similar pain relief; however, percutaneous leads have a higher risk of migration, which can lead to system failure; therefore, surgical leads should be considered as the preferred lead to implant according to our results.

In cases where percutaneous lead requires removal, surgical leads offer a viable alternative with effective pain relief. Further studies are needed to determine whether one lead type offers superior long-term outcomes, especially referring to patients who received an implanted surgical system as a rescue therapy after removal of a percutaneous system.

## Figures and Tables

**Table 2:** Demographic data at implantation.

Variable	Number	Percentage
Gender		
Male	106	56.38%
Female	82	43.62%
Age		
Minimum	15	
Maximum	76	
Less than 40 yo	35	18.62%
40 yo or more	153	81.38%
Cause of pain		
FBS	120	63.83%
CRPS	56	29.79%
Others	12	6.38%
Type of lead		
Surgical	68	36.17%
Percutaneous	120	63.83%
Lead location		
Cervical	36	19.15%
Dorsal	152	80.85%
Type of stimulation		
Tonic	153	81.38%
High frequency	21	11.17%
Change from tonic to high frequency	14	7.45%
Posterior surgeries	57	30.32%
Minimum	1	
Maximum	5	
Total	188	

**Table 3:** Analysis of minor and major complications.

Complications	Number	Percentage
Major complications	36	100%
Infections	19	52.78%
Minimum infections	1	
Maximum infections	6	
Lead migration	15	41.67%
Epidural hematoma	2	5.56%
Minor complications	68	100.00%
Unwanted electric pulse	8	11.76%
Pocket problems	36	52.94%
Impedance increase	24	35.29%

**Table 4:** Analysis of work status and reincorporation, pain relief and satisfaction.

Variable	Number	Percentage
Work status		
Active	18	11.11%
Temporal leave	47	29.00%
Other	97	59.88%
Work reincorporation		
Yes	10	6.17%
No	36	22.21%
Does not proceed	116	71.60%
PGI-C score		
Effective	140	86.42%
Non-effective	22	13.58%
Pain relief percentage		
Effective	91	56.17%
Non-effective	71	43.83%
Would implant again		
Yes	131	80.86%
Total	162	100.00%

**Table 5:** Comparison of demographic variables between surgical and percutaneous electrodes. Abbreviations: *n*, number; %, percentage; yo, years old; x, mean; sd, standard deviation.

	Surgical Lead (*n* = 68) (*n*, %) (x ± sd)	Percutaneous Lead (*n* = 120) (*n*, %) (x ± sd)	*p* Value
Sex			0.615
Male	40 (58.82%)	66 (55.00%)	
Female	28 (41.18%)	54 (45.00%)	
Age			0.898
Less than 40 yo	13 (19.12%)	22 (18.33%)	
40 yo or older	55 (80.68%)	98 (81.67%)	
Age at implantation	47.04 ± 9.17	47.03 ± 9.72	0.999
Lead location			0.054
Cervical	8 (11.76%)	28 (23.33%)	
Dorsal	60 (88.24%)	92 (76.67%)	

**Table 6:** Comparison of complications between surgical and percutaneous electrodes. Abbreviations: *n*, number; %, percentage; x, mean; sd, standard deviation.

Complications	Surgical Lead (*n* = 68) (*n*, %) (x ± sd)	Percutaneous Lead (*n* = 120) (*n*, %) (x ± sd)	*p* Value
Minor complications			0.322
Yes	25 (36.8%)	43 (35.8%)	
No	43 (63.2%)	77 (64.2%)	
Major complications			0.278
Yes	12 (17.65%)	22 (18.33%)	
No	56 (82.35%)	98 (81.67%)	
Total complications	0.61 ± 0.84	0.68 ± 0.81	0.499
Infection			0.111
Yes	10 (14.71%)	9 (7.50%)	
No	58 (85.29%)	111 (92.50%)	
Number of infections	2.6 ± 2.01	1.88 ± 1.69	0.300
Lead migration			**0.051**
Yes	2 (2.94%)	13 (10.83%)	
No	66 (97.06%)	107 (89.17%)	
Pocket problems			0.444
Yes	10 (14.71%)	23 (19.17%)	
No	58 (85.29%)	97 (80.83%)	
Impedance increase			0.545
Yes	10 (14.71%)	14 (11.67%)	
No	58 (85.29%)	106 (88.33%)	

**Table 7:** Comparison of pain relief between surgical and percutaneous electrodes. Abbreviations: *n*, number; %, percentage; x, mean; sd, standard deviation.

	Surgical Lead (*n* = 55) (*n*, %) (x ± sd)	Percutaneous Lead (*n* = 107) (*n*, %) (x ± sd)	*p* Value
PGI-C score			0.474
Effective	49 (89.09%)	91 (85.05%)	
Non-effective	6 (10.91%)	16 (14.95%)	
PGI-C score	2.49 ± 1.19	2.46 ± 1.11	0.888
Pain relief percentage			0.976
Effective	31 (56.36%)	60 (56.07%)	
Non-effective	24 (43.64%)	47 (43.93%)	
Pain relief	56.72 ± 27.75	53.92 ± 28.17	0.304
Work reincorporation			0.416
Yes	1 (1.82%)	9 (9.41%)	
No	10 (18.18%)	26 (24.30%)	
Does not proceed	44 (80.00%)	72 (67.29%)	
Would reimplant			0.297
Yes	42 (76.36%)	89 (83.18%)	
No	13 (23.64%)	18 (16.82%)	

**Table 8:** Clinical comparison between surgical and percutaneous leads in patients who have had both systems. Abbreviations: p50, 50th percentile; iqr, interquartile range.

Variable	Surgical Lead (p50 ± iqr)	Percutaneous Lead (p50 ± iqr)	*p* Value
PGI-C score	2 ± 1	3 ± 1	0.082
Pain relief percentage	70 ± 30	45 ± 30	0.035

## Data Availability

The datasets presented in this article are not readily available because of ethical reasons.

## Abbreviations

The following abbreviations are used in this manuscript:

IASP	International Association for the Study of Pain
SCS	Spinal cord stimulation
FBS	Failed back syndrome
CRPS	Complex regional pain syndrome
